# 2-(1,3-Dioxoisoindolin-2-yl)acetonitrile

**DOI:** 10.1107/S1600536810037335

**Published:** 2010-09-25

**Authors:** Younas Aouine, Anouar Alami, Abdelilah El Hallaoui, Abdelrhani Elachqar, Hafid Zouihri

**Affiliations:** a Laboratoire de Chimie Organique, Faculté des Sciences, Dhar el Mahraz, Université Sidi Mohammed Ben Abdellah, Fès, Morocco; b Laboratoires de Diffraction des Rayons X, Division UATRS, Centre National pour la Recherche Scientifique et Technique, Rabat, Morocco

## Abstract

The asymmetric unit of the title compound, C_10_H_6_N_2_O_2_, contains two independent mol­ecules. The dihedral angles between the acetonitrile and the 1*H*-isoindole-1,3(2*H*)-dione units are 69.0 (7)° and 77.0 (5)° in the two mol­ecules. One of the two terminal N atoms is disordered over two positions in a 0.66 (8):0,34 (8) ratio. In the crystal structure, the mol­ecules are linked by inter­molecular C—H⋯O hydrogen bonds.

## Related literature

The title compound was prepared as a key intermediate for the synthesis of a new new tetrazolic derivative. For the use of tetra­zoles as pesticides, see: Schocken *et al.* (1989 ▶); Yanagi *et al.* (2001 ▶); Lim *et al.* (2007 ▶) and as anti­hypertensive, anti­alergic, anti­biotic and anti­convulsant agents, see: Hashimoto *et al.* (1998 ▶); Berghmans *et al.* (2007 ▶). For their use in cancer, AIDS and obesity treatments, see: Tamura *et al.* (1998 ▶); Shih *et al.* (1999 ▶); Muraglia *et al.* (2006 ▶). A major advantage of tetra­zoles over carb­oxy­lic acids is that they are resistant to many biological metabolic degradation pathways, see: Singh *et al.* (1980 ▶).
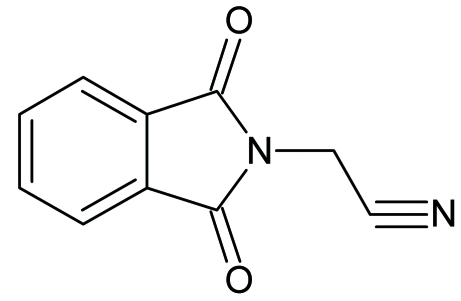


## Experimental

### 

#### Crystal data

C_10_H_6_N_2_O_2_
*M* *_r_* = 186.17
Triclinic, 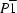

*a* = 8.0960 (2) Å
*b* = 8.4371 (2) Å
*c* = 14.3118 (3) Å
α = 85.072 (1)°
β = 79.272 (1)°
γ = 68.421 (1)°
*V* = 893.02 (4) Å^3^
*Z* = 4
Mo *K*α radiation
μ = 0.10 mm^−1^
*T* = 296 K
0.25 × 0.24 × 0.16 mm

#### Data collection

Bruker APEXII CCD detector diffractometer
18332 measured reflections
3906 independent reflections
2885 reflections with *I* > 2σ(*I*)
*R*_int_ = 0.034

#### Refinement

*R*[*F* ^2^ > 2σ(*F* ^2^)] = 0.043
*wR*(*F* ^2^) = 0.128
*S* = 1.05
3906 reflections
267 parameters
6 restraints
H-atom parameters constrained
Δρ_max_ = 0.19 e Å^−3^
Δρ_min_ = −0.20 e Å^−3^

### 

Data collection: *APEX2* (Bruker, 2005 ▶); cell refinement: *SAINT* (Bruker, 2005 ▶); data reduction: *SAINT*; program(s) used to solve structure: *SHELXS97* (Sheldrick, 2008 ▶); program(s) used to refine structure: *SHELXL97* (Sheldrick, 2008 ▶); molecular graphics: *PLATON* (Spek, 2009 ▶); software used to prepare material for publication: *publCIF* (Westrip, 2010 ▶).

## Supplementary Material

Crystal structure: contains datablocks I, global. DOI: 10.1107/S1600536810037335/jh2206sup1.cif

Structure factors: contains datablocks I. DOI: 10.1107/S1600536810037335/jh2206Isup2.hkl

Additional supplementary materials:  crystallographic information; 3D view; checkCIF report

**Fig. 3.**
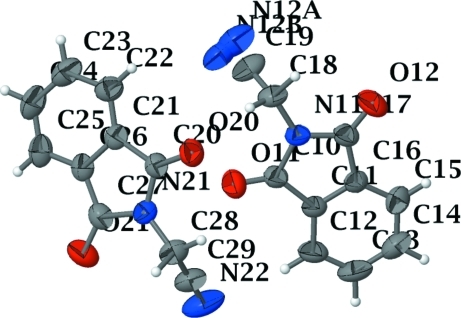
View of the title compound showing displacement ellipsoids at the 50% probability level.

Enhanced figure: interactive version of Fig. 3

## Figures and Tables

**Table 1:** Hydrogen-bond geometry (Å, °)

*D*—H⋯*A*	*D*—H	H⋯*A*	*D*⋯*A*	*D*—H⋯*A*
C13—H13⋯O12^i^	0.93	2.51	3.386 (2)	158
C15—H15⋯O20^ii^	0.93	2.45	3.144 (2)	132
C18—H18*B*⋯O20	0.97	2.42	3.372 (2)	167
C28—H28*A*⋯O21^iii^	0.97	2.39	3.298 (2)	156

Symmetry codes: (i) 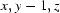; (ii) 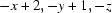; (iii) 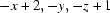.

**Fig. 1.**
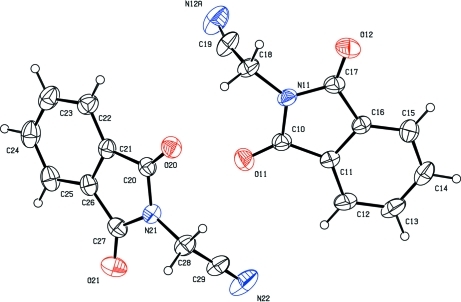
Two independent molecules of the title compound showing the atom-labelling scheme and 30% probability displacement ellipsoids. Only major parts of disordered N are shown.

**Fig. 2.**
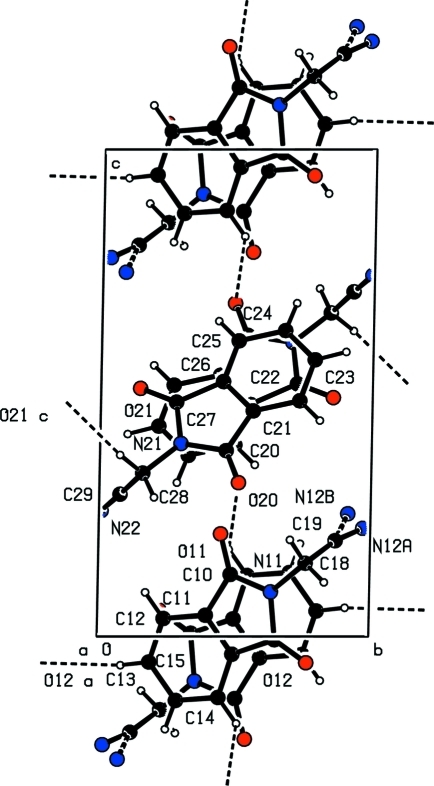
Partial packing view showing the formation of a chain through C—H···O hydrogen bonds shown as dashed lines.

## supplementary crystallographic information

### Comment

With the aim of developing new tetrazolic derived, an analog isosteric of the glycine, we have prepared 2-(1,3-dioxoisoindolin-2-yl)acetonitrile,a key intermediate, starting from 2-(bromomethyl)isoindoline-1,3-dione.

The asymmetric unit of the new synthetized 2-(1,3-dioxoisoindolin-2-yl)acetonitrile, C_10_H_6_N_2_O_2_, contains two independent molecules. The dihedral angles between the acetonitrile and the 1*H*-isoindole-1,3(2*H*)-dione are 69.0 (7)° and 77.0 (5)°, respictively.

One of the two terminal N is disordered over two positions with occupancy of 0.66 (8) for the major site. In the crystal structure, the molecules are linked by intermolecular C—H···O hydrogen bonds.

### Experimental

A mixture containing 4.8 g (0.02 mol) of 2-(bromomethyl)isoindoline-1,3-dione, 6.5 g KCN (0.1 mol), and 60 ml of anhydrous acetonitrile is heated overnight at 60°C, and then filtered. The residue is washed twice with acetonitrile, and the filtrate was concentrated under vacuum. The solid obtained is purified by chromatography on silica gel column (eluent: ether / hexane: 2 / 3).

Yield= 80% (white solid); F= 122–124°C; Rf = 0.31(ether/hexane3:1).

IR (KBr) ν cm^-1^: 3070(CH_arom_),2947/2983 (CH), 1692/1709 (2 C=O), 1557/1613 (C=C). *δ*_H_ (CDCl_3_): 4.57 (2H_CH2_,s); 7.60–8.10 (4H_arom_, m). *δ*_C_ (CDCl_3_): 28.1(CH_2_);115.01(CN); 127.5; 132.1; 133.3(C_arom_); 168.28(2 C=O).

MS—EI: [*M*]^+^=186.

Elemental analysis for C_10_H_6_N_2_O_2_ Calcd(Found):*C* 64.51(64.62), H 3.22(3.31), N 15.02(14.94).

### Refinement

All H atoms were fixed geometrically and treated as riding with C—H = 0.97 Å (methyne) and 0.93Å (aromatic) with *U*_iso_(H) = 1.2Ueq(C).

One of the terminals N was found disordered over. Two sets of positions were defined for the disordered N and the site occupation factors were refined while restraining their sum to unity. The site occupation factor of the major component was refined to 0.66 (8).

### Crystal data

**Table:**

C_10_H_6_N_2_O_2_	*Z* = 4
*M**_r_* = 186.17	*F*(000) = 384
Triclinic, *P*1	*D*_x_ = 1.385 Mg m^−^^3^
Hall symbol: -P 1	Melting point: 395 K
*a* = 8.0960 (2) Å	Mo *K*α radiation, λ = 0.71073 Å
*b* = 8.4371 (2) Å	Cell parameters from 2714 reflections
*c* = 14.3118 (3) Å	θ = 2.7–25.3°
α = 85.072 (1)°	µ = 0.10 mm^−^^1^
β = 79.272 (1)°	*T* = 296 K
γ = 68.421 (1)°	Block, colourless
*V* = 893.02 (4) Å^3^	0.25 × 0.24 × 0.16 mm

### Data collection

**Table:**

Bruker APEXII CCD detector diffractometer	2885 reflections with *I* > 2σ(*I*)
Radiation source: fine-focus sealed tube	*R*_int_ = 0.034
graphite	θ_max_ = 27.0°, θ_min_ = 1.5°
ω and φ scans	*h* = −10→10
18332 measured reflections	*k* = −10→10
3906 independent reflections	*l* = −17→18

### Refinement

**Table:**

Refinement on *F*^2^	Primary atom site location: structure-invariant direct methods
Least-squares matrix: full	Secondary atom site location: difference Fourier map
*R*[*F*^2^ > 2σ(*F*^2^)] = 0.043	Hydrogen site location: inferred from neighbouring sites
*wR*(*F*^2^) = 0.128	H-atom parameters constrained
*S* = 1.05	*w* = 1/[σ^2^(*F*_o_^2^) + (0.0697*P*)^2^ + 0.070*P*] where *P* = (*F*_o_^2^ + 2*F*_c_^2^)/3
3906 reflections	(Δ/σ)_max_ = 0.005
267 parameters	Δρ_max_ = 0.19 e Å^−^^3^
6 restraints	Δρ_min_ = −0.19 e Å^−^^3^

### Special details

**Table:**

Geometry. All e.s.d.'s (except the e.s.d. in the dihedral angle between two l.s. planes) are estimated using the full covariance matrix. The cell e.s.d.'s are taken into account individually in the estimation of e.s.d.'s in distances, angles and torsion angles; correlations between e.s.d.'s in cell parameters are only used when they are defined by crystal symmetry. An approximate (isotropic) treatment of cell e.s.d.'s is used for estimating e.s.d.'s involving l.s. planes.
Refinement. Refinement of *F*^2^ against ALL reflections. The weighted *R*-factor *wR* and goodness of fit *S* are based on *F*^2^, conventional *R*-factors *R* are based on *F*, with *F* set to zero for negative *F*^2^. The threshold expression of *F*^2^ > σ(*F*^2^) is used only for calculating *R*-factors(gt) *etc*. and is not relevant to the choice of reflections for refinement. *R*-factors based on *F*^2^ are statistically about twice as large as those based on *F*, and *R*- factors based on ALL data will be even larger.

### Fractional atomic coordinates and isotropic or equivalent isotropic displacement parameters (Å^2^)

**Table:**

	*x*	*y*	*z*	*U*_iso_*/*U*_eq_	Occ. (<1)
C11	0.73565 (18)	0.39842 (17)	0.04371 (10)	0.0471 (3)	
C16	0.75085 (18)	0.49910 (17)	−0.03635 (10)	0.0461 (3)	
C10	0.7395 (2)	0.48752 (18)	0.12747 (11)	0.0497 (3)	
C17	0.7711 (2)	0.65409 (18)	−0.00671 (11)	0.0527 (4)	
C18	0.7772 (2)	0.76260 (19)	0.15037 (12)	0.0582 (4)	
H18A	0.8360	0.8327	0.1112	0.070*	
H18B	0.8503	0.7049	0.1984	0.070*	
C15	0.7474 (2)	0.4505 (2)	−0.12523 (12)	0.0612 (4)	
H15	0.7559	0.5195	−0.1788	0.073*	
C14	0.7309 (2)	0.2948 (2)	−0.13128 (13)	0.0685 (5)	
H14	0.7291	0.2577	−0.1903	0.082*	
C19	0.5995 (3)	0.8711 (2)	0.19668 (15)	0.0745 (5)	
C12	0.7182 (3)	0.2438 (2)	0.03696 (14)	0.0670 (5)	
H12	0.7075	0.1754	0.0906	0.080*	
C13	0.7170 (3)	0.1937 (2)	−0.05234 (15)	0.0750 (5)	
H13	0.7065	0.0893	−0.0589	0.090*	
C20	0.91639 (18)	0.46404 (18)	0.38170 (10)	0.0462 (3)	
C26	0.72827 (18)	0.4498 (2)	0.52338 (10)	0.0495 (3)	
C21	0.78905 (18)	0.56105 (19)	0.46334 (10)	0.0472 (3)	
C27	0.81143 (19)	0.2782 (2)	0.48113 (11)	0.0512 (4)	
C28	1.0390 (2)	0.1584 (2)	0.33559 (12)	0.0598 (4)	
H28A	1.1202	0.0741	0.3727	0.072*	
H28B	1.1114	0.2015	0.2857	0.072*	
C22	0.7336 (2)	0.7317 (2)	0.48390 (12)	0.0600 (4)	
H22	0.7740	0.8068	0.4433	0.072*	
C29	0.9423 (2)	0.0764 (2)	0.29222 (14)	0.0661 (5)	
C23	0.6146 (2)	0.7856 (2)	0.56814 (14)	0.0715 (5)	
H23	0.5744	0.8996	0.5843	0.086*	
C25	0.6098 (2)	0.5045 (3)	0.60702 (12)	0.0640 (4)	
H25	0.5686	0.4297	0.6475	0.077*	
C24	0.5549 (2)	0.6749 (3)	0.62815 (13)	0.0725 (5)	
H24	0.4758	0.7154	0.6842	0.087*	
O11	0.72249 (19)	0.44907 (16)	0.21083 (8)	0.0739 (4)	
O12	0.7926 (2)	0.77161 (16)	−0.05386 (10)	0.0863 (4)	
O20	1.00395 (15)	0.51155 (14)	0.31593 (8)	0.0613 (3)	
O21	0.79522 (16)	0.14499 (15)	0.50949 (9)	0.0727 (4)	
N11	0.76467 (16)	0.63694 (14)	0.09160 (9)	0.0497 (3)	
N21	0.92041 (16)	0.29721 (15)	0.39635 (8)	0.0494 (3)	
N22	0.8731 (3)	0.0080 (2)	0.25846 (16)	0.1020 (7)	
N12A	0.4599 (15)	0.972 (4)	0.222 (2)	0.100 (4)	0.66 (8)
N12B	0.466 (4)	0.916 (6)	0.252 (3)	0.085 (6)	0.34 (8)

### Atomic displacement parameters (Å^2^)

**Table:**

	*U*^11^	*U*^22^	*U*^33^	*U*^12^	*U*^13^	*U*^23^
C11	0.0471 (8)	0.0391 (7)	0.0555 (8)	−0.0166 (6)	−0.0054 (6)	−0.0037 (6)
C16	0.0411 (7)	0.0434 (7)	0.0506 (8)	−0.0148 (6)	0.0017 (6)	−0.0064 (6)
C10	0.0539 (8)	0.0439 (8)	0.0529 (9)	−0.0215 (6)	−0.0049 (6)	−0.0009 (6)
C17	0.0550 (8)	0.0447 (8)	0.0597 (9)	−0.0227 (7)	−0.0037 (7)	0.0018 (7)
C18	0.0611 (9)	0.0469 (8)	0.0728 (10)	−0.0233 (7)	−0.0141 (8)	−0.0108 (7)
C15	0.0567 (9)	0.0693 (10)	0.0519 (9)	−0.0200 (8)	0.0035 (7)	−0.0102 (8)
C14	0.0631 (10)	0.0669 (11)	0.0705 (11)	−0.0135 (8)	−0.0062 (8)	−0.0307 (9)
C19	0.0700 (12)	0.0728 (12)	0.0872 (14)	−0.0258 (10)	−0.0134 (10)	−0.0353 (10)
C12	0.0875 (12)	0.0422 (8)	0.0784 (12)	−0.0294 (8)	−0.0189 (9)	0.0008 (8)
C13	0.0835 (12)	0.0453 (9)	0.1006 (15)	−0.0209 (9)	−0.0214 (11)	−0.0213 (9)
C20	0.0446 (7)	0.0517 (8)	0.0462 (8)	−0.0201 (6)	−0.0124 (6)	0.0007 (6)
C26	0.0418 (7)	0.0632 (9)	0.0449 (8)	−0.0185 (7)	−0.0123 (6)	0.0011 (7)
C21	0.0432 (7)	0.0540 (8)	0.0462 (8)	−0.0167 (6)	−0.0127 (6)	−0.0035 (6)
C27	0.0455 (8)	0.0578 (9)	0.0532 (8)	−0.0209 (7)	−0.0143 (6)	0.0073 (7)
C28	0.0518 (9)	0.0554 (9)	0.0701 (10)	−0.0151 (7)	−0.0088 (8)	−0.0125 (8)
C22	0.0573 (9)	0.0558 (9)	0.0674 (10)	−0.0164 (7)	−0.0166 (8)	−0.0072 (8)
C29	0.0628 (10)	0.0485 (9)	0.0830 (12)	−0.0116 (8)	−0.0123 (9)	−0.0185 (8)
C23	0.0590 (10)	0.0699 (11)	0.0766 (12)	−0.0041 (8)	−0.0181 (9)	−0.0257 (10)
C25	0.0492 (9)	0.0898 (13)	0.0496 (9)	−0.0219 (8)	−0.0069 (7)	−0.0001 (8)
C24	0.0517 (9)	0.0973 (15)	0.0572 (10)	−0.0114 (9)	−0.0049 (8)	−0.0202 (10)
O11	0.1064 (10)	0.0764 (8)	0.0524 (7)	−0.0494 (8)	−0.0150 (6)	0.0068 (6)
O12	0.1289 (12)	0.0668 (8)	0.0810 (9)	−0.0601 (8)	−0.0181 (8)	0.0195 (7)
O20	0.0651 (7)	0.0686 (7)	0.0530 (6)	−0.0320 (6)	−0.0011 (5)	0.0014 (5)
O21	0.0695 (7)	0.0627 (7)	0.0869 (9)	−0.0291 (6)	−0.0133 (6)	0.0184 (6)
N11	0.0584 (7)	0.0396 (6)	0.0556 (7)	−0.0229 (5)	−0.0072 (6)	−0.0052 (5)
N21	0.0499 (7)	0.0496 (7)	0.0498 (7)	−0.0186 (5)	−0.0072 (5)	−0.0065 (5)
N22	0.0928 (13)	0.0766 (11)	0.1438 (18)	−0.0253 (10)	−0.0265 (12)	−0.0498 (12)
N12A	0.078 (2)	0.098 (7)	0.114 (8)	−0.013 (4)	−0.013 (4)	−0.048 (7)
N12B	0.079 (5)	0.081 (10)	0.088 (10)	−0.020 (6)	0.005 (5)	−0.040 (7)

### Geometric parameters (Å, °)

**Table:**

C11—C12	1.376 (2)	C20—N21	1.3944 (18)
C11—C16	1.381 (2)	C20—C21	1.481 (2)
C11—C10	1.480 (2)	C26—C21	1.380 (2)
C16—C15	1.378 (2)	C26—C25	1.381 (2)
C16—C17	1.482 (2)	C26—C27	1.482 (2)
C10—O11	1.2052 (18)	C21—C22	1.382 (2)
C10—N11	1.3903 (18)	C27—O21	1.2066 (17)
C17—O12	1.1989 (18)	C27—N21	1.3958 (19)
C17—N11	1.395 (2)	C28—N21	1.4445 (19)
C18—N11	1.4511 (18)	C28—C29	1.460 (2)
C18—C19	1.458 (2)	C28—H28A	0.9700
C18—H18A	0.9700	C28—H28B	0.9700
C18—H18B	0.9700	C22—C23	1.388 (2)
C15—C14	1.380 (2)	C22—H22	0.9300
C15—H15	0.9300	C29—N22	1.125 (2)
C14—C13	1.369 (3)	C23—C24	1.372 (3)
C14—H14	0.9300	C23—H23	0.9300
C12—C13	1.383 (3)	C25—C24	1.383 (3)
C12—H12	0.9300	C25—H25	0.9300
C13—H13	0.9300	C24—H24	0.9300
C20—O20	1.2021 (17)		
			
C12—C11—C16	120.65 (14)	C21—C26—C27	108.31 (13)
C12—C11—C10	130.71 (15)	C25—C26—C27	130.42 (15)
C16—C11—C10	108.64 (12)	C26—C21—C22	121.60 (14)
C15—C16—C11	121.80 (13)	C26—C21—C20	108.40 (13)
C15—C16—C17	130.20 (14)	C22—C21—C20	129.99 (14)
C11—C16—C17	108.00 (13)	O21—C27—N21	124.00 (15)
O11—C10—N11	123.96 (14)	O21—C27—C26	130.39 (15)
O11—C10—C11	130.57 (14)	N21—C27—C26	105.61 (12)
N11—C10—C11	105.46 (12)	N21—C28—C29	112.91 (13)
O12—C17—N11	124.60 (15)	N21—C28—H28A	109.0
O12—C17—C16	129.75 (15)	C29—C28—H28A	109.0
N11—C17—C16	105.63 (12)	N21—C28—H28B	109.0
N11—C18—C19	111.30 (13)	C29—C28—H28B	109.0
N11—C18—H18A	109.4	H28A—C28—H28B	107.8
C19—C18—H18A	109.4	C21—C22—C23	116.75 (17)
N11—C18—H18B	109.4	C21—C22—H22	121.6
C19—C18—H18B	109.4	C23—C22—H22	121.6
H18A—C18—H18B	108.0	N22—C29—C28	177.51 (18)
C16—C15—C14	117.07 (16)	C24—C23—C22	121.71 (17)
C16—C15—H15	121.5	C24—C23—H23	119.1
C14—C15—H15	121.5	C22—C23—H23	119.1
C13—C14—C15	121.47 (16)	C26—C25—C24	117.30 (17)
C13—C14—H14	119.3	C26—C25—H25	121.4
C15—C14—H14	119.3	C24—C25—H25	121.4
C11—C12—C13	117.66 (16)	C23—C24—C25	121.36 (16)
C11—C12—H12	121.2	C23—C24—H24	119.3
C13—C12—H12	121.2	C25—C24—H24	119.3
C14—C13—C12	121.34 (16)	C10—N11—C17	112.21 (12)
C14—C13—H13	119.3	C10—N11—C18	123.66 (13)
C12—C13—H13	119.3	C17—N11—C18	124.11 (12)
O20—C20—N21	124.84 (14)	C20—N21—C27	112.00 (12)
O20—C20—C21	129.50 (14)	C20—N21—C28	123.18 (12)
N21—C20—C21	105.65 (12)	C27—N21—C28	124.49 (13)
C21—C26—C25	121.28 (15)		

### Hydrogen-bond geometry (Å, °)

**Table:**

*D*—H···*A*	*D*—H	H···*A*	*D*···*A*	*D*—H···*A*
C13—H13···O12^i^	0.93	2.51	3.386 (2)	158
C15—H15···O20^ii^	0.93	2.45	3.144 (2)	132
C18—H18B···O20	0.97	2.42	3.372 (2)	167
C28—H28A···O21^iii^	0.97	2.39	3.298 (2)	156

Symmetry codes: (i) *x*, *y*−1, *z*; (ii) −*x*+2, −*y*+1, −*z*; (iii) −*x*+2, −*y*, −*z*+1.
